# Euthanasia as Medical Therapy in Canada

**DOI:** 10.1002/hast.70004

**Published:** 2025-09-11

**Authors:** Trudo Lemmens

**Keywords:** euthanasia, assisted suicide, assisted dying, capacity, informed consent, medical ethics, health policy

## Abstract

*This commentary argues that recent reports of an Ontario coroner's office's MAiD Death Review Committee confirm how Canada's euthanasia regime has normalized ending of life as a form of therapy, often for only indirectly health‐related suffering. The author, a member of the committee, illustrates with some of the cases how access to death rather than protection against premature death appears to be prioritized, often after very basic capacity and informed consent procedures by health professionals with limited training in relevant end‐of‐life health care*.

## Perspective

Less than a decade after Canada legalized euthanasia and assisted suicide as medical assistance in dying (MAID), lethal injections have ended around 70,000 lives. About 5 percent of all deaths in Canada are by MAID, and in Quebec, the figure is 7.2 percent, the highest euthanasia rate of any jurisdiction worldwide.

For ardent MAID supporters, this is a success story in which offer meets consumer demand. Critics see growing concerns. In 2025, the United Nations Committee on the Rights of Persons with Disabilities called on Canada to cancel its 2021 expansion of MAID eligibility beyond people approaching their death and to halt further expansion, citing, among other evidence, 2024 reports of the MAiD Death Review Committee (MDRC) of the Office of the Chief Coroner for Ontario, which revealed practices that supporters denied would ever happen. (I am a member of this committee.) A 2024 report on MAID and social and structural inequities documents a young woman's receipt of MAID for lack of affordable housing that met her accessibility needs (to address the medical condition of multiple chemical sensitivity). Other cases reveal providers’ failures to explore underlying socioeconomic reasons for MAID requests and instances of people struggling with untreated mental illness.

Take the case of a man in his forties with inflammatory bowel disease, a history of suicidal ideation, and unaddressed substance use who learned about MAID from a psychiatrist. The MAID provider noted family “concerns” about his MAID request but found him eligible and “transported him to an external location for the provision of MAiD.”

An MDRC report on the practice of “same day and next day provisions” reveals remarkable fast‐tracking of euthanasia. One elderly woman declined MAID and preferred palliative care. When a hospice placement request was rejected, her husband, who had been assessed as struggling with “caregiver burnout” asked for an urgent MAID assessment. She was euthanized that day.

Cases often reveal perplexing capacity and informed consent procedures. A palliative care team considered a man, who had requested a MAID consult but had become delirious and was sedated, no longer capable of decision‐making. Yet the MAID provider “vigorously roused” him, the provider and a second assessor accepted “mouth[ing] ‘yes’” and nodding as expressions of informed consent, and he was euthanized that day.

The cases illustrate one of the most pernicious consequences of Canada's euthanasia regime: the normalization of health care providers’ ending of life as a therapy, often for only indirectly health‐related suffering. Though this death therapy is aggressively promoted as medical practice, no clear diagnostic criteria specify when it is warranted. Access criteria are vague. The Canadian Association of MAID Assessors and Providers, an advocacy organization with disproportionate influence on MAID policy and practice, issued guidance that straddles the contours of legality. Nearly any chronic illness can be forced to fit access criteria, even when its etiology is not understood and experts still explore treatments. CAMAP even provides guidance to overcome a longer evaluation period for individuals not approaching their death: for example, a patient's stated intent to, in the future, refuse antibiotic treatment for a serious infection makes death “foreseeable,” thus enabling same‐day MAID.

The rigorous evidence‐informed clinical standards one expects of new medical specialties are absent. Frequent providers dominate the practice (in Canada in 2023, 361 providers performed 10,138 of 15,343 procedures). Their original training often has little to do with end‐of‐life care or relief of suffering or with the chronic conditions for which they are now providing MAID. And physicians must ensure access to MAID even if other options for relief of suffering remain—with Canada's MAID, patient choice trumps standard therapy.

Yet, because MAID is sold as medicine, problems similar to those in ordinary practice arise. The undervaluing of disabled people's quality of life appears to influence how providers deem “choice” as inherently autonomous. And motivations that sometimes drive medical malpractice may influence MAID. A lethal injection pays better than a palliative care consult. It may also be a convenient practice. One provider, Stefanie Green, declared “delivering at the end of life” easier to schedule than delivering babies, her original specialty.

When it comes to, say, overprescription of opioids, regulators fall back on agreed‐upon medical standards to determine misconduct. But MAID criteria are vague and open‐ended; standards, ill‐defined. Little wonder no one has so far been held criminally accountable for what remains a criminal‐code exemption for murder and aiding suicide.

